# Tuberculoma-Induced Seizures

**DOI:** 10.5811/westjem.2015.7.27758

**Published:** 2015-10-20

**Authors:** R. James Salway, Shruti Sangani, Samira Parekh, Sanjay Bhatt

**Affiliations:** * Keck School of Medicine of USC, Department of Emergency Medicine, Los Angeles, California; † BJ Civil Medical Center, Ahmedabad, Gujarat, India

## Abstract

Seizures in human immunodeficiency virus (HIV) patients can be caused by a wide variety of opportunistic infections, and, especially in developing countries, tuberculosis (TB) should be high on the differential. In India, TB is the most common opportunistic infection in HIV and it can have several different central nervous system manifestations, including intracranial tuberculomas. In this case, an HIV patient presenting with new-onset seizure and fever was diagnosed with tuberculous meningitis and multiple intracranial tuberculomas. The patient received standard TB medications, steroids, and anticonvulsants in the emergency department and was admitted for further care.

## INTRODUCTION

While traditionally thought of as a primarily pulmonary process, tuberculosis (TB) can affect a variety of organ systems, including the central nervous system (CNS). CNS manifestations include tuberculous meningitis (TBM), spinal tuberculous arachnoiditis, and intracranial tuberculomas.^7^ Intracranial tuberculomas are understood to be caused by hematagenous spread of bacillus into the brain, establishing tubercles that can coalesce and grow. Tuberculomas can exhibit as a single large mass or as multiple masses throughout the brain, and are more likely to be found in the posterior fossa.^12^

Tuberculomas are of growing clinical importance as ever-increasing globalization leads to increased migration and expansion of TB. The World Health Organization estimates there are approximately nine million new cases of TB each year, with over 20% of these cases demonstrating extrapulmonary disease.^9^ Intracranial tuberculomas are currently relatively rare in the Western world, comprising approximately 0.15%–0.18% of all brain tumors and are found largely in adults suffering from TB reactivation.^5^ Tuberculomas are significantly more common in the developing world and are estimated to compromise of 20%–30% of all brain masses^1^,^5^ They are also more common in children and associated with concurrent human immunodeficiency virus (HIV) infection.^12^ TB is the most common cause of death in HIV-infected patients, and approximately 24% of all TB deaths worldwide are associated with HIV.^4^,^14^

## CASE REPORT

A patient was brought in by relatives to the emergency department (ED) of the BJ Civil Medical Center in the city of Ahmedabad in Gujurat, India. The patient and his family complained of a single generalized tonic-clonic seizure, altered mental status, and four episodes of emesis throughout the day. Upon further history, the patient and his relatives detailed a history of HIV diagnosed in 2003. The patient had been prescribed anti-retroviral therapy (ART) with Efavirenz and Lamivudine. However, he had been non-compliant with his medication for the past two weeks. His last CD4 count checked two years ago was 276. On review of systems, the patient also complained of a fever and a headache for the last 1.5 months for which he had not sought medical attention.

On presentation to the ED, the patient’s vital signs were a blood pressure of 112/80mmHg, a pulse of 108 beats per minute, a respiratory rate of 20 breaths/min and an oxygen saturation of 99% on room air. His temperature was 102 degrees Fahrenheit. On physical exam, the patient was confused and irritable; he was alert and oriented to person only. His neurological exam failed to demonstrate any localized neurological deficit, with a normal Babinski reflex and no neck rigidity. The patient’s ophthalmologic/fundal exam demonstrated no papillaedema. His lungs were clear to auscultation bilaterally. In the ED, the patient did not have any seizure activity.

While the patient’s laboratory results were pending, he was started on prophylactic treatment of meningitis with intravenous ceftriaxone 2 grams, vancomycin 1 gram. Additionally, he was treated with ondansetron 4 milligrams, phenytoin 1 gram, and acetaminophen 325 milligrams.

The patient’s blood work demonstrated a complete blood count with a white blood count of 11,070/mm^3^ and no neutrophil predominance, and a hemoglobin 12.5 grams. Additional pertinent laboratory results included an erythrocyte sedimentation rate of 70mm/hr and a CD4 count of 59. The patient’s liver function tests were normal. The patient’s chest radiograph did not demonstrate any evidence of pulmonary infiltrates, cavitations, or consolidations.

The patient underwent a magnetic resonance imaging (MRI), which was concerning for tuberculous leptomeningitis with multiple tuberculomas in the left occipital parasagittal region with enhancement of infective granulomatous tissue in the left sylvian fissure (Figure). A lumbar puncture was performed and cerebrospinal fluid (CSF) examination revealed elevated protein (162mg/dL), low levels of glucose (33mg/dL), and a total cell count of 90/mm^3^ with an 80% lymphocyte predominance.

The patient was determined to have TBM and intracranial tuberculomas. He was started on isoniazid 10mg/kg daily, rifampin 10mg/kg daily, pyrazinamide 35mg/kg daily, streptomycin 15mg/kg intramuscular 3 times per week, and pyridoxine 50mg daily. He was also started on intravenous steroids: dexamethasone 8mg every eight hours for three days and then switched to prednisolone 40mg daily. The patient additionally received prophylactic trimethoprim/sulfamethoxazole and valproic acid.

On day 3, the patient defervesced; his mental status returned to baseline on day 5. He was restarted on ART on day 7 and discharged home on day 8.

## DISCUSSION

The workup for an HIV positive patient who presents with altered mental status often focuses on more common etiologies, including toxoplasmosis, bacterial or fungal meningitis, or neurocystercercosis. In the West, intracranial tuberculomas are an uncommon cause of seizure. These tuberculomas often remain clinically silent until they exhibit a mass effect on the brain and present as seizures, headaches, gait disturbances, and visual fields defects.^5^,^7^ These patients often have a normal neurological exam, though papillaedema can be present if the intracranial pressure is sufficiently elevated. Sixth nerve palsies are the most frequently appreciated neurological deficit.^12^

Patients with intracranial tuberculomas often lack a history of TB infection or conversely may even be on TB medications at the time of presentation.^5^,^12^ As countries with the most prevalent cases of TB often administer the BCG vaccine, a PPD at the time of presentation is not traditionally very helpful.^5^ The presence of active pulmonary TB on chest radiograph ranges from 30 to 50% in one series.^10^

Diagnostic imaging is important when investigating the possibility of a CNS TB infection. A computed tomography of the head with intravenous contrast is often sufficient. Tuberculomas appear as an avascular low-density mass lesion. Often, they will exhibit greater than expected surrounding cerebral edema.^5^,^7^ Late-stage tuberculomas are well encapsulated and have peripheral ring enhancement. This can lead to a common misdiagnosis of neurocystercercosis, especially if multiple tuberculomas are present.^7^ An MRI of the brain is also a useful adjunct imaging modality in distinguish between the two entities.

Generally, lumbar punctures should be avoided as the space occupying mass of the tuberculoma can theoretically cause herniation^5^,^7^ Furthermore, CSF results are often unremarkable-although in our patient, the CSF did demonstrate an elevated protein level, leukocytosis, and a decreased glucose level.^5^

The treatment for intracranial tuberculomas is predominantly medical. There are several case series of surgical interventions for confirmed tuberculomas with relatively high mortality and increased risk for severe meningitis following surgical excision.^7^,^12^,^13^ Surgery may be warranted in cases with concern for obstructed hydrocephalus, compression of the brainstem, or impending herniation.^7^,^12^

First-line medications for intracranial tuberculomas mirror those for TB meningitis. Isoniazid, rifampin, and pyrazinamide all have adequate CSF penetration and are bactericidial.^7^ Ethambutol and streptomycin are considered second line on account of their poor CNS penetration and their adverse effects. In the case of intracranial tuberculomas, the duration of treatment doubles to 18 months. Given the prolonged length of treatment, the physician should remain alert to side effects including isoniazid-induced hepatitis and neurotoxicity.^5^,^7^

Corticosteroids also play a prominent role in the medical treatment of intracranial tuberculomas. Many of the symptoms of intracranial tuberculomas are secondary to increased intracranial pressure from the disproportionate cerebral edema caused by the lesions. In randomized control trials, dexamethasone and prednisone have both been shown reduce cerebral edema and reduce mortality in TB meningitis.^3^,^8^,^11^ They play a similar role in intracranial tuberculomas and are strongly indicated.^5^,^7^

Upon diagnosis, tuberculomas carry a positive prognosis. All the literature examined showed complete or near-complete recovery for all patients on medical treatment, and neither surgically nor medically treated patients had any recurrence after 28 months.^1^,^5^,^12^

## CONCLUSION

In an immunocompromised patient who presents with seizures, especially if from an area with endemic TB, a physician should consider the diagnosis of intracranial tuberculomas. Computed tomography (CT) imaging remains the diagnostic modality of choice, and a lumbar puncture should be withheld until a space-occupying lesion has been ruled out and a physician looks to evaluate for TB meningitis. Treatment in the ED should focus on anti-seizure medications, standard anti-TB regimens with isoniazid, rifampin, and ethambutol, and early corticosteroid administration with dexamethasone or prednisone. Surgical intervention should be reserved to patients with signs of obstructive hydrocephalus or brainstem compression. Response to treatment can be followed by repeat CT imaging to visualize decreased cerebral edema and reduction in intracranial tuberculoma size. The disease process carries a good prognosis.

As TB and HIV/acquired immune deficiency syndrome continue to affect a larger geographic area and international travel becomes easier and more prevalent, a high index of suspicion is required to diagnose and treat intracranial tuberculomas.

## Figures and Tables

**Figure:**
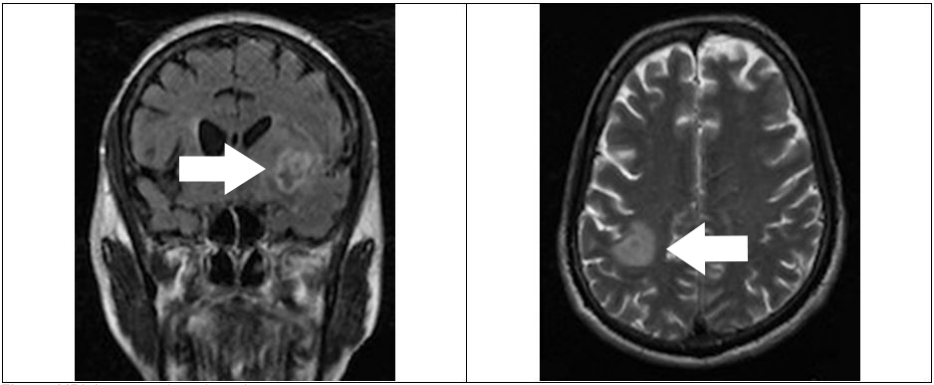
MRI demonstrating tuberculoma. *MRI*, magnetic resonance imaging
